# Surveying Parents of Preschool Children about Digital and Analogue Play and Parent–Child Interaction

**DOI:** 10.3390/children10020251

**Published:** 2023-01-30

**Authors:** Andreja Istenič, Violeta Rosanda, Mateja Gačnik

**Affiliations:** 1 Faculty of Education, University of Primorska, Cankarjeva 5, 6000 Koper, Slovenia; 2 Institute of Psychology and Education, Kazan Federal University, Kremlyovskaya 18, 420000 Kazan, Russia; 3 Center for the Communication, Hearing and Speech Portorož, Sončna pot 14a, 6320 Portorož, Slovenia

**Keywords:** digital play, analogue play, parent–child interaction, communication, parental mediation, toddlers

## Abstract

The addition of digital toys to the child’s toy box has resulted in the development of the new ‘digital play’, which differs from analogue play. Research shows that digital toys are available from infancy onwards and are significantly changing the way children engage in play and communicate with parents during play. How this influences the child’s development must be established. The choice of toys and the manner in which they are used depend greatly on the parents. In the present study, parents’ opinions and experiences of their child’s digital and analogue play were explored in order to gain insight into the parents’ perceptions of the impact of different types of play on their child’s development. We were particularly interested in the differences in a child’s engagement with a toy and the child–parent interaction and communication. In this descriptive study, we administered a questionnaire in order to collect data from 306 parents of children of an average age of 3.6 years. The results show that parents perceived traditional toys as the most stimulating toys for a toddler’s sensory, motor, cognitive, and socio-emotional development. During analogue play, significantly more parent–child interaction, as well as more language input from parents and toddlers, occurred. Parents also used different intervention and mediation strategies with different types of toys.

## 1. Introduction

Digital technology has become an integral part of our lives and has changed the communication practices between people, reflecting the need to develop new skills for effective communication [1,2]. With the entry of technology into homes, which are the environments in which children develop [2], the way they interact with adults and their play behaviors [3,4] have changed.

The challenge confronting parents is to provide an appropriate environment and experiences that are stimulating for the child’s development and to also prepare the child to communicate effectively in the modern world. On one hand, parents are concerned about the negative impact that technology may have on the child’s development, and on the other hand, they are concerned about failing to meet the social expectations that new technology brings [5]. The proliferation of digital technologies has reached children’s toys and has raised concern about what constitute child-appropriate digital toys. Digital play and digital toys, which have characteristics of virtual versus tangible, with affordances for interaction and manipulation, must be examined and presented to parents.

The child’s environment is constructed through societal processes utilizing physical and digital materialities. The proliferation of digital technologies is affecting interaction and socialization, as well as the perception of reality (materiality of physical and digital and transmedia practices), and the child’s agency. Furthermore, the parent–child interaction process takes place in an environment that is constituted by digital technology. During the earliest period of life, the parents’ interaction with the child, in which non-verbal communication and eye contact play an integral role, is important. We were interested in how, according to parents, the inclusion of traditional and electronic toys affects both verbal and non-verbal communication. That is, we sought to determine how parents would explain a child’s behavior and interaction during play using electronic versus traditional means and the effects of a child’s interaction in these different contexts [6].

A child is born into a cultural environment that is marked by cultural tools, and his/her play using these cultural tools is the primary mode of cultural development [7]. According to Vygotsky [7], the child’s development of higher psychological functions, such as language, takes place in the communication with the parents in the child’s primary environment, which is the home. The child’s interaction and communication with the parents occur through play involving materiality, including digital toys, screen-based digital play, and digital devices. Play manifests socio-cultural practices and inclusive practices involving digital technology. The technological environment is ubiquitous and enters various social practices seamlessly [8].

“Research demonstrates that developmentally appropriate play with parents and peers is a singular opportunity to promote the social-emotional, cognitive, language, and self-regulation skills that build executive function and a prosocial brain” [9] (p. 2). The process of playing provides a transition from reactive action to cognitive comprehension and the play process, as such, supports the development of artefacts and concepts [7]. A child uses artefacts in the proximate environment to undertake locomotor play (exercise play) and social play during the early stage of life. Play provides parents with the convenience to engage with children using toys as a medium of play and interaction [3]. 

Technology has transformed the home environment, raising the question of its impact on a child’s development. On the one hand, a technology-rich environment is reflected in the increased use of digital technology within the home environment and on the other hand it is changing the way children play. The studies investigating the impact of the changed environment show benefits [10,11] as well as negative effects [12,13,14,15], such as technoference and a disturbance in parent–child communication [14,15]. In addition, the new toy box, which includes more and more different toys in addition to the traditional ones, brings changes to children’s play. Digital play consists of digital screen toys, digital non-screen toys [16], and digital screen technology for online platforms providing software and applications to facilitate a child’s play. We classify toys [17] into categories of traditional toys (e.g., plush toys, dolls, puzzles, and all other toys that do not run on batteries, electricity, or solar power); simple electric and electronic toys without screens (battery-powered, electricity-powered, or solar-powered, but not involving computer technology); digital non-screen toys (toys with computer technology but without a screen, some of which can be connected to the Internet); digital screen toys (toys with computer technology and a screen that run on batteries, electricity, or solar power); and digital screen technology with a screen that is not primarily made for play but can be used for play (e.g., smartphone, tablet, laptop, PC).

### 1.1. Digital Play and Child Development

The changes that have been brought by digital play have raised concerns about its impact on children’s development. Electronic media exposure was found to affect the behavioral, cognitive, and socio-affective development of children [18]. Digital play with electronic media and toys has been associated with lower amounts of cognitive, language, socio-emotional, and gross motor activity, and has correlated with childhood obesity and developmental issues [19,20,21]. Based on a systematic review, Bocchiccio and colleagues [22] point out that the nature of digital play is more complex and can result in positive, as well as negative, effects on a child’s cognitive, social, and emotional development. Focusing on child language development, adult–child communication is recognized as its essential component [23,24], where the appropriate linguistic input of the adult and the activity of the child are important. The quality and the quantity of language have been positively connected to children’s language outcomes [25]. Understanding the impact of the changed environment in which the child and the parent interact on the child’s language development is crucial. Digital technology, with television being among the first examples, was found to cause a reduction in parent–child interaction. Christakis and colleagues [19] examined audible television and its influences on interaction patterns and identified decreases in the child’s exposure to adult speech and the child’s vocalization. Similar results were found in studies examining play with digital toys as opposed to traditional toys. A reduced quality of parental and child language, as well as a reduction in the child’s vocalization and conversational turn-taking during play with digital toys, were found [4,20,26,27,28]. In terms of the quantity of parental language, the results have been mixed, showing the presence of equal [20] or less parental language [4,26,27]. Regarding language quality and interaction, Zosh and colleagues [20] argued that the design of toys can seriously affect parents’ play style, the toys’ use, and the language that this elicits. 

Parents’ opinions regarding the impact of technology on the child’s development, as well as their preferences of traditional or digital toys, are important because of their key role in shaping the home environment and play. Family values were found to have the greatest impact on the accessibility and rules regarding the use of technology, its acquisition, and the relationship between technological and digital toys and the activities of children [29]. The studies on young children’s parent’s beliefs and attitudes toward child digital exposure show mixed results, with some finding a belief of a negative effect or a concern of a potentially negative effect of technology on the child’s health and physical development, as well as social interaction [29], and others finding positive attitudes and beliefs [30]. Research shows that parents prefer traditional toys over digital toys for their children [31], even when taking into account different cultures [32].

### 1.2. Parent–Child Interactions during Play

According to Nathanson [33], in addition to the amount or frequency of use, the qualitative aspect, or how parents and children use technology, must be taken into account, as the method of use can reduce the negative effects. Active engagement, as well as parents’ mediation strategies [34], were found to trigger more positive effects.

Different types of interactions can take place during the use of digital media. The classification of parental mediation strategies follows the trends that have been brought about by a technologically rich environment and the needs of the researchers who study the impact of a changed environment on various aspects of child development. Three mediation strategies were proposed by Valkenburg and colleagues [35] that were designed to research television mediation, including (1) restrictive mediation, where the parent sets time or content restrictions or prohibitions; (2) instructive mediation, where the parent discusses the content with the child; and (3) social co-viewing, where the programming is watched together. The fourth strategy, which is known as participatory learning, where the interaction between parents and children occurs via digital media, was added by Clark [36]. The co-viewing strategy is the least encouraging for interactions as it takes place mostly non-verbally [37] and signals parental approval of the media content and the media practice [38]. Restrictive mediation was found to lead to less exposure to media risks [39], but it may cause the opposite of the desired effects [40,41]. Joint media engagement was found to be conducive to the child’s language development [40] and has been associated with instructive mediation and participatory learning. Studies concerning the impact of joint media engagement on children’s language development have shown mixed results, with some uncovering non-significant outcomes in children’s language development [42,43] and others finding a negative impact [44,45]. Research has shown that parents use different types of mediation strategies [34,36,46,47] depending on their beliefs about digital technology [6,8]. The differences in the mediation method that is used are in accordance with the parents’ positive or negative expectations of the digital media’s effects on their children [34], cultural orientation [34,48], motive [49], own media use and skills, and family context variables [35]. The increased societal focus on academic readiness also affects parents’ practices in encouraging the use of structured activities that are designed to promote academic results in preschool [9,49]. Regarding the influence of the child’s age and gender on parental mediation, studies have revealed differences that are related to the child’s age group [50,51], but not to child’s gender [51,52], especially in younger children. The studies of older children in pre-adolescence and adolescence reveal some differences in the mediation strategies of parents of girls and boys [53,54]. Parental mediation changes as the child gets older, with instructional intermediation being more common in younger children [50]. Parents’ mediation of children’s digital media use is increasingly important due to the presence of digital technology at younger and younger ages [50,55]. Parental concerns about the potential negative effects of technology encourage restrictive mediation, while a healthy level of concern influences more active mediation strategies [34,50], which, according to the research so far, yield better results. Parental contribution to and influence on a child’s language development is essential and it is conducted through play using toys as mediators. Digital toys enter the home environment, and the extent to which the parents will productively use them in play with a child depends on the parents’ beliefs about the potential of digital technology and digital toys for the child’s development and learning. As parents shape the home environment, it is important to investigate their position regarding the impact of digital technologies on children’s learning and play. The purpose of this descriptive research is to seek answers to two rudimental research questions and to check the hypotheses supporting each research question regarding children’s gender differences.

**RQ1.**  How do parents perceive the roles of digital and analogue play in a child’s cognitive, sensory, psychophysical, and socio-emotional development? How do they initiate the use of digital technology and toys? Is there a difference according to the child’s gender?

**H1.**  *Parents perceive the role of digital and analogue play and a child’s environment to be the same, regardless of the child’s gender.*

**RQ2.**  How do parents perceive the interaction-communication space when the child engages with different types of toys? How do they communicate and interact with a child during play? Is there a difference according to the child’s gender?

**H2.**  *How parents perceive the interactive-communicative space when the child engages with different types of toys and how they communicate and interact with the child during play is the same, regardless of the child’s gender.*

## 2. Materials and Methods 

### 2.1. Research Design and Instruments

A survey was conducted among parents of toddlers. The descriptive study examined the Slovene parents’ opinions and perceptions of play and how they structure their child’s play by offering them a set of toys. 

The questionnaire design was based on a literature review [3,4,30,56,57,58,59,60,61,62,63,64,65]. The first part consisted of descriptive questions about the child and the parent (child age, child gender, child position in the family, parent role, parent gender, parent age, living area, and the number of children in the family).

The second part consisted of questions about play and toys (traditional, simple non-screen electric, and electronic toys; digital non-screen toys and digital screen toys; digital technology; and non-digital objects and artefacts), digital technology and apps, and the child’s daily activity. Toy ownership and the frequency of digital technology offered to a child are also part of this article. The types of toys included a classification of toys that the authors made on the basis of the literature review [17]. The parents were asked to assess the amount of playtime in percentages. They could select from the following range of toy types: traditional toys (toys not running on batteries, electricity, or solar power); simple electric and electronic toys without screens (battery-powered, electricity-powered, or solar-powered toys, but not involving computer technology); digital non-screen toys (toys with computer technology but without a screen, some of which can be connected to the Internet); digital screen toys (toys with computer technology and a screen that run on batteries, electricity, or solar power); digital screen technology with a screen that is not primarily designed for gaming but can be used for gaming (smartphone, tablet, laptop, PC, etc.); and non-digital objects and artefacts.

The third part solicited the parents’ opinions about digital play’s contribution to learning and development and their perception of the child’s play. The main part of the results of the second and third parts are presented in an article by Istenič and colleagues [66].

The fourth part focused on the contribution of the different types of toys to areas of learning and development (indicating sensory, motor, cognitive, and emotional development) [61] and examined the interaction-communication space that the child engages during play and the skill development when playing with different types of toys. A set of statements indicated the level of attention, readiness, and engagement of a child whether observing, listening, touching, or verbally expressing and talking to a toy or to a person. The fourth part is the focus of this article. 

In this section, the types of toys were matched (yes/no) with statements indicating their contribution to different areas of learning and development, the child’s play behaviors and interaction, and the parent’s mediation in play.

### 2.2. Sample

The survey gathered a non-randomized sample of 277 mothers (90%) and 29 fathers (10%). The parents reported on their child’s behalf; 48% were girls and 52% were boys, and their average age was 3.6 years. The children’s age range includes those from the age of 1 to the age of 5.

### 2.3. Data Collection and Analysis

Data were collected with an online questionnaire from March to May 2021. Before starting the questionnaire, the parents agreed to written consent regarding anonymity, aggregated data analysis, and reporting.

Data analysis was conducted with SPSS. The reliability of the instrument was tested with Cronbach alpha, which indicated the sufficient reliability of the instrument with the Cronbach alpha between 0.79 and 0.92. We conducted data analysis by applying descriptive and inferential statistics. The data are presented in structural tables indicating percentage frequencies (f%), mean with standard deviation, and the chi-square test is applied to check the hypotheses.

## 3. Results

First, we examined the parents’ views on the impact of the child’s play with digital and analogue toys on his/her cognitive, sensory, psychophysical, and socio-emotional development, and how they introduce digital technology and toys to their child. The parents rated the child’s activities in play with analogue toys in contrast to digital toys, how the child interacts with different types of toys, the accompanying parent–child interaction, and the parent’s intervention. In the results, the presentation starts with toy ownership and how the parents initiate the use of digital technology to their child.

### 3.1. Parental Assessment of Their Child’s Toy Ownership and Initiation of Digital Technology

The parents assessed the share of the toy types that their children own, which were listed in a question. As presented in Table 1, traditional toys dominate among the toys that are owned by Slovenian children, accounting for a mean of 71% of all toys, with girls having a higher mean (M = 74.6%, SD = 26.78) vs. boys (M = 67.5%, SD = 20.50). At second place is the category of digital technology with computer screen technology that is not primarily designed for play (M = 21.1%), with boys having a higher mean (M = 10.3%, SD = 21.76) vs. girls (M = 9.9, SD = 20.22).

In third place is the category of simple non-screen electric or electronic toys (M = 19.2%), with boys having a higher mean (M = 22%, SD = 21.2) vs. girls (M = 16.3%, SD = 17.03). The least represented categories are the digital non-screen toys (M = 15.6%), with boys having a higher mean (M = 7.1 SD = 17.22) vs. girls (M = 5.4, SD = 14.50), and the digital screen toys (M = 14.2%), with boys having a slightly higher mean (M = 4.9, SD = 15.22) vs. girls (M = 4.9, SD = 14.34). The chi-square test results indicated gender difference in a child’s toy ownership for all types of toys and digital technology, traditional toys (χ^2^ 347.154; g = 56; *p* = 0.001), simple non-screen electric and electronic toys (χ^2^ 338,752; g = 56; *p* = 0.001), digital non-screen toys (χ^2^ 332.714; g = 34; *p* = 0.001), digital screen toys (χ^2^ 329.100; g = 32; *p* = 0.001), and computer screen technology (χ^2^ 332.384; g = 36; *p* = 0.001). The hypothesis stating that there is no gender difference can be rejected.

In order to examine how parents initiate the use of digital technology to their child, the parents presented how often they offer digital devices to their child. As shown in a Table 2, smart phone is most often offered to a child (M = 2.2, SD = 1.06), followed by a tablet (M = 1.6, SD = 0.92). A PC is offered less often (M = 1.03, SD = 0.6). The means of boys and girls are almost similar, and the chi-square results indicated no gender difference; therefore, the devices are not offered in relation to a child’s gender.

### 3.2. Parents’ Perception of the Roles of Digital and Analogue Play on Child’s Development

In order to obtain data related to the first research question, in which we wanted to gain insight into parents’ opinions regarding the impact of traditional and digital games on children’s development, we asked the parents to state their opinion regarding the contribution of each type of toy to their child’s development. In the questionnaire, the parents marked yes/no for the types of toys for which they agreed with the statement.

The parents’ evaluations of the impact of the different types of toys on their child’s development and play are presented in Table 3. In the questionnaire, the parents marked ‘yes’ or ‘no’ for the types of toys for which they agreed with the statement. Relatively few parents did not give an opinion on a certain item and chose the option of ‘I do not know’ (between 2% and, at most, 9% on the item that was related to emotional development), which means that parents have a developed opinion regarding the impact of each type of toy on their child’s development. The parents considered the traditional toys to be the most beneficial toys for their child’s development because they are the most stimulating for sensory, motor, cognitive, and emotional development, as well as for listening and observation, and visual and spatial orientation. Most of the parents perceived the traditional toys as being able to stimulate sensory development (94%), motor development (93%), emotional development (87%), and cognitive development (70%). The traditional toys were also recognized as the most encouraging in the area of listening and observation (74%), as well as visual and spatial orientation (84%). The parents perceived all of the other types of toys as contributing significantly less to the child’s development across all areas of enquiry. The digital screen toys and technology were perceived by over 40% of the parents as being able to enhance cognitive development and listening and observation, but they were rated significantly lower on all of the other items (19% of the parents recognized their contribution to visual and spatial orientation, 17% to sensory development, 15% to emotional development, and 6% to motor development). Similarly, but with a slightly lower percentage (just over 30%), parents rated the electric and electronic toys as having a favorable impact on cognitive development and listening and observation. Their impact on sensory development and visual and spatial orientation was recognized by 22% of the parents. According to the parents, electric and electronic toys make the smallest contribution to children’s motor (16%) and emotional (11%) development. The lowest rating was given to the digital non-screen toys, which parents did not perceive as particularly stimulating in any of the development areas that were listed. The highest percentage of parents, which was still low compared to the whole (22%), rated them as stimulating for cognitive development, listening and observation (20%). The estimated contribution to sensory development (11%), visual and spatial orientation (11%), and motor development (3%), are the lowest valued, with a minority of parents recognizing their benefit.

The chi-square tests identified a significant gender difference in assigning how toy types contribute to different areas of learning and development for only one type of toy in three instances, which was the digital non-screen toys and their contribution to emotional development (χ^2^ = 7.543; g = 1; *p* = 0.006), listening and observing (χ^2^ = 4.963; g = 1; *p* = 0.026), and visual and spatial orientation (χ^2^ = 5.143; g = 1; *p* = 0.023). For these three instances, the hypothesis stating that there is no gender difference can be rejected.

### 3.3. A Child’s Interaction with a Toy and Communication with Parents during Play

Research question two deals with the way in which children engage in interaction with the toy and how they communicate with parents. In order to gain insight into this area of interest, data on three questions of the questionnaire were analyzed. The results are presented below. In the questionnaire, the parents marked yes/no for the types of toys for which they agreed with the statement. 

Table 4 presents the results regarding the child’s engagement when playing with different types of toys. The parents expressed the belief that when they are playing with traditional toys, the child is interested in interacting with the play partner (92%), plays independently with the toy (92%), shows attention to the game and the toy (91%), initiates play with the toy (91%), and interacts intensively with the toy (89%). Regarding the predominant sensory activity during play with traditional toys, most of the parents identified toy manipulation (82%). Observing and listening were rated as the dominant activities by 45% and 29% of parents, respectively. According to the parents, in terms of attention to play and toys, the electric and electronic toys (41%), as well as the screen-based digital toys and technology (39%), scored significantly lower than the traditional toys (91%), but significantly better than the non-screen-based digital toys (18%). A similar relationship between the different types of toys was found in the child’s expression of interest in communication with the adult or peer play partner. Most of the parents noticed that the child expresses an interest in communicating with the play partner while playing with traditional toys (92%), while significantly less parents noticed the latter feature when playing with electric and electronic toys (26%), digital screen toys and technology (15%), and finally non-screen toys (9%). In terms of independence and the child’s initiative, the electric and electronic toys hold the second place, after the traditional ones, with 36% of parents recognizing independence and 35% recognizing the child’s initiative while playing with this type of toy. In third place are the digital toys, which were recognized by a slightly smaller percentage of the parents as evoking the child’s initiation (29%) and independence (27%) while playing. The lowest number of parents rated their child’s independence (15%) and initiation (12%) when playing with digital non-screen toys. As the predominant sensory activity of play, the parents identified different ones, depending on the type of toy. When they are playing with traditional toys, the parents recognized that the child mostly manipulates (82%), when playing with digital screen toys and technology, the child watches (58%), and when they are playing with electric or electronic toys and digital non-screen toys, the child listens (32%, 26%). The parents reported watching (58%) and listening (51%) as dominant in their toddler’s play with digital toys and technology, which is consistent with the reported impact of these toys on listening and observation development, where digital screen toys and technology ranked second only to traditional toys. Among the listed activities, the digital non-screen toys scored last in terms of the frequency of responses on all of the items.

The chi-square test results indicated a gender difference for children’s engagement in play in only two instances and for two types of toys, as follows: the digital non-screen toys when the child predominantly watches (χ^2^ = 10.352; g = 4; *p* = 0.035) and electric and electronic toys when the child intensively communicates with a toy (χ^2^ = 9.920; g = 4; *p* = 0.042). For these two instances, the hypothesis can be rejected.

The parents’ evaluations of the parent–child interactions accompanying play with different toy types are shown in Table 5. In the questionnaire, the parents marked yes/no for the types of toys for which they agreed with the statement.

Similar to the results that have been shown previously, the traditional toys proved to be the most suitable for quality parent–child interaction according to the parents. The parents reported that during play with traditional toys, the communication between them and their toddler was the most intense (92%), with frequent physical contact (90%) and eye contact (89%), the toddler talking most frequently (89%), as well as seeking to be near the parents (80%) and to attract their attention (86%). They also addressed the child more frequently (87%) and focused more on the child than on the toy (84%). On all of the mentioned items, all of the other types of toys received a significantly lower frequency of agreement from the participating parents. In addition to the traditional toys, the effect of which was recognized by the parents on all of the items that were related to active play, a larger number of the parents (20% or more) also recognized the value of some other types of toys. Thus, in terms of causing the least tension and conflict, the digital screen toys and technology came in second place, with 24% of the parents agreeing with the statement. In terms of the children’s more frequent vocalization or speech, the electric toys ranked second, with 26% of the parents agreeing with the statement. Regarding the expression of joy during play, the parents recognized it in their child’s play with digital screen toys and technology (26%), as well as with the electric and electronic toys (25%). However, passive play was most often observed with digital screen toys or technology (56%).

The traditional toys were found to be most suitable for expressing emotions, with the majority of parents associating them with the toddler’s most frequent expressions of emotion (89%) and joy (88%) during play. There was also the least amount of child–parent conflict during play with the traditional toys.

The parents reported that their toddlers played for the longest with the traditional toys, followed by the digital screen toys and technology. While 76% of the parents recognized that their toddlers sustained their play with traditional toys for a long time, 39% reported lengthy play with digital screen toys or technology as well. The chi-square test results indicated no gender difference for parent interaction with the child in play.

The parents reported using different strategies for mediating their toddler’s play. In the questionnaire, the parents marked ‘yes’ or ‘no’ for the types of toys for which they agreed with the statement. Table 6 presents the results of the parents’ behavior during the toddler’s play with the different types of toys. The parents were asked to present strategies during the period of one month before the questionnaire. Regarding the amount of mediation in different types of play, the parents reported most frequently mediating the child’s digital play with digital screen toys and digital apps. They used significantly less mediation for play with the electric and electronic toys and play on different types of consoles.

As for the type of mediation used, the parents reported co-viewing and restriction strategies as the most commonly used types of mediation. Co-viewing was typically associated with mediating television (57%) and digital media use (51%). Similarly, the parents reported the use of instructive mediation mostly in digital media use (42%), followed by television viewing (31%). Many of the parents also reported using instructive mediation strategies in order to actively mediate play through discussion for digital media (42%) and television (31%) use. Participatory learning was reported as the least common mediation strategy in TV and digital media use. The parents reported encouraging digital media use by joining (24%) and intervening in the game to help (34%). Similar results were found for television use. Interestingly, when the child played with the digital toys without screens and with the electric and electronic toys, the parents mediated the least, but when they did mediate, they used participatory learning strategies.

The chi-square test results indicated no gender difference for parental mediation in the child’s play.

## 4. Discussion

In this study, we gathered parents’ opinions and experiences of toddlers’ digital and analogue play in order to determine the parents’ perceptions of the contribution of digital and analogue play to a child’s cognitive, sensory, psychophysical, and socio-emotional development (research question one) and how the parent–child interactions, especially communication, differ (research question two).

Hassinger-Das and colleagues [31] found that digital play is often already present in infants’ play, but when choosing between different types of toys, parents prefer traditional ones. Our study on toddlers shows similar results. The traditional toys predominated among all of the toys that were owned by the child, which was consistent with the parents’ choice as the main makers of the home environment. Children have all types of toys at home and consequently engage in analogue, as well as digital, play activities. The gender difference was established for all of the toy categories ownership in the sample that was under examination. Boys’ means are much higher compared with girls for the categories of owning simple electric and electronic toys and digital non-screen toys. Girls have a much higher mean for the traditional toys.

The caregivers’ beliefs about toy provision responding to research question one is important in shaping children’s play behaviors [31,50]. Parents’ perceptions of the contribution of toys to their child’s development are important for toy choice and use. While research has primarily focused on recommendations and observing the differences in children’s play and interaction with adults, less is known about the parents’ beliefs that also influence their behavior [66]. Studies have shown electronic media and toys to be associated with lower amounts of cognitive, language, and gross motor activity, as compared to traditional toys [19,20]. Our research, in accordance with prior studies, shows that the parents perceived traditional toys as the most stimulating toys for their child’s sensory, motor, cognitive, and emotional-behavioral skills. Their evaluation of traditional toys significantly outperformed that of all of the other types of toys on all of the items that were presented. An analysis of the gender difference in the parental perception of how toy types contribute to different areas of learning and development has been established for only one type of toy in three instances, as follows: the digital non-screen toys and their contribution to emotional development (χ^2^ = 7.543; g = 1; *p* = 0.006), listening and observing (χ^2^ = 4.963; g = 1; *p* = 0.026), and visual and spatial orientation (χ^2^ = 5.143; g = 1; *p* = 0.023). For these three instances, the hypothesis stating that there is no gender difference can be rejected.

In addressing research question two, we report children’s engagement with a toy and their interaction with an accompanying parent. Regarding children’s activities in terms of their engagement and interaction with a toy that accompany digital versus analogue play, a gender difference was indicated for children’s engagement in play, in only two instances and for two types of toys, as follows: the digital non-screen toys when a child predominantly watches (χ^2^ = 10.352; g = 4; *p* = 0.035) and electric and electronic toys when a child intensively communicates with a toy (χ^2^ = 9.920; g = 4; *p* = 0.042). For these two instances the hypothesis stating that there is no gender difference can be rejected.

The results of our study support research showing that analogue play with traditional toys leads to significantly more parent–child interactions compared to digital play [26,27,28] and a better quality of interactions [4]. In addition, and in line with some previous studies [20,26,44], parents also reported more language input from them, as well as the child. The parents recognized the interaction accompanying the digital game, as well as the fact that both their and the child’s language as poorer. There was also a difference in the mediation methods that were used during play. Parents were more likely to use restrictive mediation and co-viewing in connection with the child playing with screen technology, whereas they were more likely to mediate with participatory learning strategies in play with the electric and electronic toys, as well as the digital non-screen toys. We found no gender difference for parent interaction with a child in play.

Our results show that the type of toy also influences the frequency and mediation technique that is used by the parent. Active mediation techniques are important, especially in digital play, as they can have a mitigating effect on the negative influences [50]. Therefore, the rate of use of mediation techniques by parents is particularly worrying. This result may be related to the sample being numerically dominated by mothers, as research (e.g., [34]) has found differences in mediation strategies between mothers and fathers. In addition, for parental mediation in a child’s play, there was no gender difference established, which is consistent with previous research of the age group that was under consideration here [51,52].

The limitations of our study arose from the non-randomized sample, which allowed us to interpret data and draw conclusions from this sample. We suggest that the conclusions of this study should be used for planning future studies on a child’s primary environment within the family. Parents have the important role of leading a child’s communication and language development. Observations of play and parent–child interactions utilizing digital toys as a medium of interaction are necessary in order to establish the affordance of a range of digital toys. As understanding parents’ perceptions of the educational affordance of digital toys is essential and will facilitate parents’ decision making, parental attitudes and beliefs should be examined. This descriptive study supports starting the process of exploration and hypothesizing about the parents’ perceptions of play, the role of digital play, and the potential effects on parent–child communication. Parents’ perceptions contribute to their decisions on which toys to offer to their children.

In future research, it would be interesting to assess which toys children are more likely to use, what the ratio between their digital and analogue play is, and also the consequences of the initiation of the use of digital toys and tools. However, this is not an isolated act; it integrates socio-cultural patterns in family life, which relates to a wider societal context. The research should address all stakeholders; parents and kindergarten teachers have to establish a partnership in their efforts to provide quality experiences for a child. The socio-economic requirements in society demand an outcome-competency-oriented preschool curriculum for the set of skills and competences that are required in a labor market. Opposed to these are the concerns of the pediatric profession, indicating threats to a child’s healthy development [67]. Teachers and other pedagogical workers (pedagogues, psychologists, speech and language therapists, etc.) will have to be supported with research results addressing good practices and quality digital resources for children.

## 5. Conclusions

The aims of our study were to set two fundamental research questions in order to examine parents’ opinions and experiences of their child’s digital and analogue play and to gain insight into the parents’ perceptions of the impact of different types of play on the child’s development. Parents reported toy ownership and how much they expose their children to digital technology. A significant difference was found for all types of toys, with girls having a higher mean in traditional toy ownership, while boys have a significantly higher mean in simple electric and electronic toys, as well as higher means in all other types of toys. The parents’ assessment of their child’s engagement with different types of toys and child–parent interaction and communication is most interesting. In the area of a child’s engagement with a toy, in terms of forming an interaction and communication space around the types of toys, there was mainly no gender difference established; however, there were some exceptions for the simple electronic and electric toys in a few instances.

The findings of our study support related research showing that analogue play with traditional toys initiates more parent–child interactions with more language input and more quality interaction than digital play. We found no gender difference for parent interaction and parental mediation.

## Figures and Tables

**Table 1:** Toy ownership—analogue vs. digital.

Type of Toy	Mean	Standard Deviation
Traditional toys	71	24.77
Boys	67.5	20.50
Girls	74.6	26.78
Simple non-screen electric and electronic toys	19.2	20.62
Boys	22	21.2
Girls	16.3	17.03
Digital non-screen toys	6.2	16.54
Boys	7.1	17.22
Girls	5.4	14.50
Digital screen toys	4.8	14.83
Boys	4.9	15.22
Girls	4.7	14.34
Computer screen technology	10.3	21.59
Boys	10.6	21.76
Girls	9.9	20.22

**Table 2:** How often parents offer digital technology to a child.

Digital Device	Mean	Standard Deviation
Smart phone	2.2	1.06
Boys	2.2	1.06
Girls	2.1	1.03
Tablet	1.6	0.92
Boys	1.6	0.92
Girls	1.6	0.92
PC	1.3	0.6
Boys	1.3	0.6
Girls	1.3	0.6
Other digital technology	1.4	0.77
Boys	1.6	0.99
Girls	1.3	0.66

**Table 3:** Children’s learning and development in play—analogue vs. digital.

Items	Percentage Frequencies				
	Traditional Toys	Electric and Electronic Toys	Digital Non-Screen Toys	Digital Screen Toys and Technology	I Do Not Know
Sensory development (e.g., strengthens visual, auditory, and tactile skills)	94%	22%	11%	17%	2%
Motor development (e.g., encourages the child to move, sit, climb, stand up, and walk; promotes hand–eye coordination)	93%	16%	3%	6%	2%
Cognitive development (e.g., supports the development of speech, sounds, first words, and language skills; contributes to learning the alphabet, numbers, names of objects, etc.)	70%	32%	22%	45%	3%
Emotional development (e.g., develops the ability to manage one’s own emotions; empathy)	87%	11%	6%	15%	9%
Listening and observation	74%	37%	20%	43%	3%
Visual and spatial orientation	84%	22%	11%	19%	8%

**Table 4:** Child’s engagement in play—analogue vs. digital.

Items	Percentage Frequencies				
	Traditional Toys	Electric and Electronic Toys	Digital Non-Screen Toys	Digital Screen Toys and Technology	I Do Not Know
The child demonstates attention to the game and the toy.	91%	41%	18%	39%	2%
The child interacts intensively with the toy.	89%	25%	9%	16%	5%
The child plays with the toy independently.	92%	36%	15%	27%	2%
The child takes the initiative to play with the toy.	91%	35%	12%	29%	2%
When playing with the toy, the child expresses interest in communicating with the adult or peer who is beside him/her.	92%	26%	9%	15%	2%
The child mainly watches.	45%	22%	14%	58%	6%
The child mainly listens.	29%	32%	26%	51%	8%
The child predominantly manipulates (touches, grasps).	82%	24%	9%	15%	6%

**Table 5:** Parent–child interaction accompanying play—analogue vs. digital.

Statements	Percentage Frequencies				
	Traditional Toys	Electric and Electronic Toys	Digital Non-Screen Toys	Digital Screen Toys and Technology	I Do Not Know
... you and your child agree mostly on the focus of the game	87%	14%	3%	10%	6%
... there is low tension and conflict between you and your child	70%	15%	9%	24%	11%
... you are more focused on the child than the toy	84%	13%	6%	9%	10%
... physical contact is most frequent	90%	11%	3%	6%	6%
… eye contact is most frequent	89%	17%	6%	8%	5%
… vocal or verbal communication between you and your child is most intense	92%	15%	4%	8%	3%
... the child is most often talking, making sounds, or vocalising (but not crying or screaming)	89%	26%	8%	15%	4%
… you address the child most frequently	87%	12%	4%	13%	6%
… the child most ferquently seeks to be near you	80%	13%	5%	10%	15%
… the child most often expresses emotions	89%	13%	3%	17%	8%
… the child most frequently seeks your attention	86%	16%	4%	8%	8%
... the child most often expresses joy	88%	25%	10%	26%	4%
... the child most often passively observes	22%	13%	10%	56%	18%
... the child continues playing for a long time	76%	18%	10%	39%	4%

**Table 6:** Parental mediation in their child’s play—analogue vs. digital.

Items	Percentage Frequencies					
	Phone, Ipad, Tablet, Digital Screen Toys	Games on Different Types of Consoles	Electric and Electronic Toys	Digital Non-Screen Toys	TV	They Did Not Use Any of the Following
Watch the child	51%	3%	10%	6%	57%	19%
Restricted	51%	6%	7%	5%	39%	28%
Intervene in the game to help	34%	5%	12%	7%	33%	28%
Discuss a topic or content	42%	5%	4%	4%	31%	40%
Encourage play by joining	24%	3%	14%	9%	26%	42%

## Data Availability

The data of the present study is not openly available, as the participants did not agree to their data being shared publicly during the data collection phase of this study.
